# Profiles of movement speed and positional variability based on extended LQG for various noises

**DOI:** 10.1038/s41598-022-17485-5

**Published:** 2022-08-03

**Authors:** Yoshiaki Taniai

**Affiliations:** Graduate School of Engineering, University of Fukui, 3-9-1 Bunkyo, Fukui, 910-8507 Japan

**Keywords:** Computational neuroscience, Motor control

## Abstract

Stochastic optimal control has been studied to explain the characteristics of human upper-arm reaching movements. The optimal movement based on an extended linear quadratic Gaussian (LQG) demonstrated that control-dependent noise is the essential factor of the speed-accuracy trade-off in the point-to-point reaching movement. Furthermore, the extended LQG reproduced the profiles of movement speed and positional variability. However, the expected value and variance were computed based on the Monte Carlo method in these studies, which is not considered efficient. In this study, I obtained update equations to efficiently compute the expected value and variance based on the extended LQG. Using the update equations, I computed the profiles of simulated movement speed and positional variability for various amplitudes of noises in a point-to-point reaching movement. The profiles of movement speed were basically bell-shaped for the noises. The speed peak was changed by the control-dependent noise and state-dependent observation noise. The positional variability changed for various noises, and the period during which the variability changed differed with the noise type. Efficient computation in stochastic optimal control based on the extended LQG would contribute to the elucidation of motor control under various noises.

## Introduction

Upper-arm reaching movement is one of the most common movements studied for examining motor control. Stochastic optimal control has been studied to explain the characteristics of human upper-arm reaching movement. The studies of stochastic optimal control are based on the assumption that the reaching movement is determined to optimize an evaluation function in the presence of conceivable noise that affects the neurophysiological processing of the living body. In the reaching movement, the speed profile of the hand is bell-shaped^1^, and the positional variability has the highest value around the middle of the movement, decreasing thereafter^2–5^. In addition, the trade-off between speed and accuracy of the movement is well known^6^.

Harris and Wolpert proposed the minimum variance model^7^. The noise considered in the minimum variance model is signal-dependent noise that depends on the magnitude of the signal and is also known as multiplicative noise. The signal-dependent noise increases with the amplitude of the control signal and is called control-dependent noise. The minimum variance model is the minimization of total positional variance in the post-movement duration. The trajectory based on the minimum variance model reproduces the trajectory and speed profile of human upper-arm reaching movement. Todorov proposed the extended linear quadratic Gaussian (LQG) framework to examine stochastic optimal control and sensorimotor estimation. The extended LQG is a framework that can efficiently solve optimal feedback control under biologically conceivable noise, which is not only signal-dependent noise but also signal-independent noise, state-dependent noise, and state-independent noise^8^. The signal-independent noise is the noise that does not depend on the magnitude of the signal and is also called additive noise. In the framework, the movement dynamic system consists of state noise, which is the state-independent noise that does not depend on the magnitude of the state, as well as control-dependent noise. The feedback system consists of the state-dependent and state-independent observation noise, and the state estimation system consists of the state-independent noise, called internal estimation noise (for more details of noises, refer to “Framework of extended LQG” in the section “Methods”). The optimal movement based on the extended LQG demonstrated that control-dependent noise is the essential factor of the speed-accuracy trade-off and that the optimal speed profile is bell-shaped. In addition, the extended LQG can reproduce the profile of positional variability^5,9^. Furthermore, aspect ratio, surface area, and orientation of variability ellipses in the movement end vary with the direction under state-dependent observation noise^9^, and these characteristics have been reported in human movements^10^. On the other hand, a novel motor learning paradigm that varies the state-dependent observation noise of visual feedback in the limbs was developed to test the optimal hybrid feedforward and feedback controller based on the extended LQG^11^. The controller reproduced the human-adapted complex reaching trajectory. However, in the studies mentioned above about the extended LQG, the expected value and variance were computed based on the Monte Carlo method. The Monte Carlo method computes the expected value by repeating many trials and requires much computation time. It is important to efficiently obtain the expected value and variance to investigate whether the optimal feedback control can reproduce the characteristics of human movement in environments related to the effects of various noises.

In this study, I obtained update equations to efficiently compute the expected value and variance based on the extended LQG. Using the update equations, I computed the profiles of simulated movement speed and positional variability for various amplitudes of noises in a point-to-point reaching movement. In the computations of simulated movements, control-dependent noise is always considered, while the other noise is considered from the state-dependent observation noise, state noise, state-independent observation noise, and internal estimation noise.

## Results

**Figure 1:**
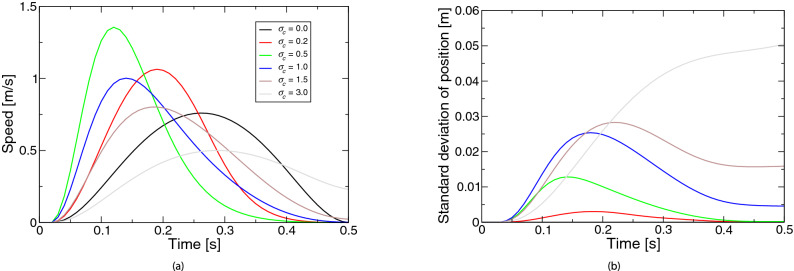
Simulated movements based on the extended LQG for the coefficient $\sigma _c$ of control-dependent noise: (**a**) speed, (**b**) positional variability. The legend of the subfigure (**b**) is the same as the subfigure (**a**).

Figure 1 represents the simulated movements based on the extended LQG for the coefficient $\sigma _c$ of the control-dependent noise. Figure 1a shows the speed profiles. The profiles were almost bell-shaped. The speed peak was about half the movement duration when the noise was absent (i.e., $\sigma _c = 0$). Although the peak appeared earlier when the noise was slight ($\sigma _c = 0.2, 0.5$), and when the noise was larger ($\sigma _c = 1.0, 1.5, 3.0$), the peak appeared later. When the noise was further larger ($\sigma _c = 3.0$), the velocity at the movement’s end was far from zero. Figure 1b shows the profiles of positional variability. When the noise was absent ($\sigma _c = 0$), the standard deviation of the position was zero (i.e., the line was identical with the *X*-axis). When the noise was larger, the variability peak appeared later and the positional variability was larger over the movement. When the noise was even larger ($\sigma _c = 3.0$), the peak disappeared (i.e., the variability increased monotonically). The optimal movement when $\sigma _c$ was 1.5 was the result for the basic parameter set. In the basic parameters, only the control-dependent noise was considered. The profiles of movement speed and positional variability resembled the characteristics reported by past studies^2–4^.

**Figure 2:**
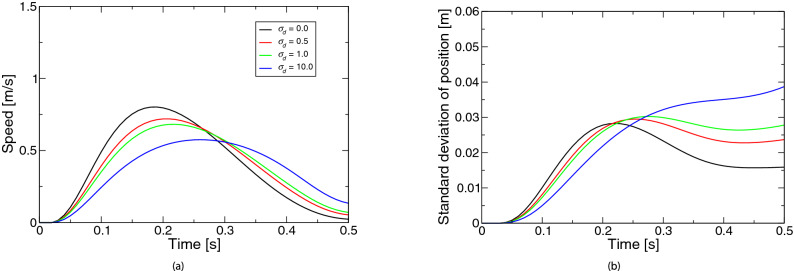
Simulated movements based on the extended LQG for the coefficient $\sigma _d$ of state-dependent observation noise: (**a**) speed, (**b**) positional variability. The legend of the subfigure (**b**) is the same as the subfigure (**a**).

Figure 2 represents the simulated movements based on the extended LQG for the coefficient $\sigma _d$ of the state-dependent observation noise. Figure 2a shows the speed profiles. All profiles were almost bell-shaped. As the noise became larger, the speed peak appeared later. Figure 2b shows the profiles of positional variability. Unlike the case of control-dependent noise, no significant change occurred up to about half of the movement, although the variability was larger in the latter half of the movement.

**Figure 3:**
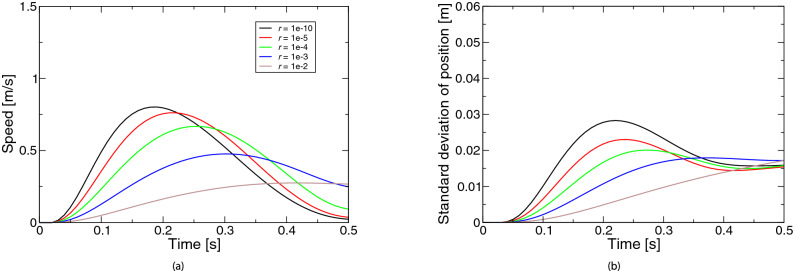
Simulated movements based on the extended LQG for the coefficient *r* of control cost: (**a**) speed, (**b**) positional variability. The legend of the subfigure (**b**) is the same as the subfigure (**a**).

Figure 3 represents the simulated movements based on the extended LQG for the coefficient *r* of the control cost. Figure 3a shows the speed profiles. As the control cost was larger, the speed peak appeared later. Figure 3b shows the profiles of positional variability. Unlike the case of state-dependent observation noise, there was a large change up to about half of the movement but little change after half the movement. In other words, as the ratio of state cost increased, the variability around the middle of the movement became larger.

**Figure 4:**
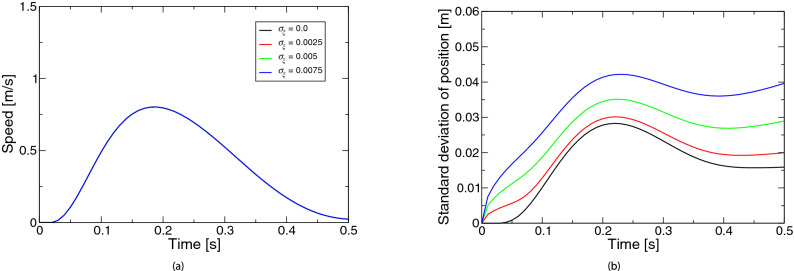
Simulated movements based on the extended LQG for the coefficient $\sigma _\xi$ of state noise: (**a**) speed, (**b**) positional variability. The legend of the subfigure (**b**) is the same as the subfigure (**a**).

**Figure 5:**
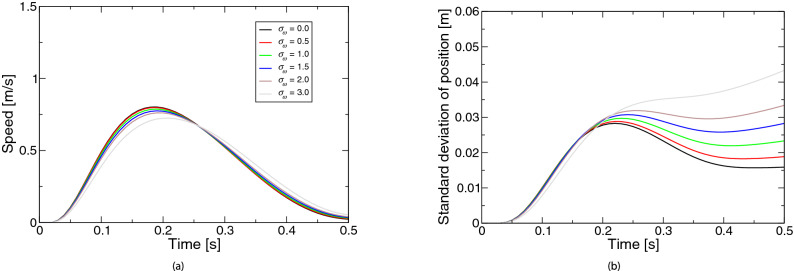
Simulated movements based on the extended LQG for the coefficient $\sigma _\omega$ of state-independent observation noise: (**a**) speed, (**b**) positional variability. The legend of the subfigure (**b**) is the same as the subfigure (**a**).

**Figure 6:**
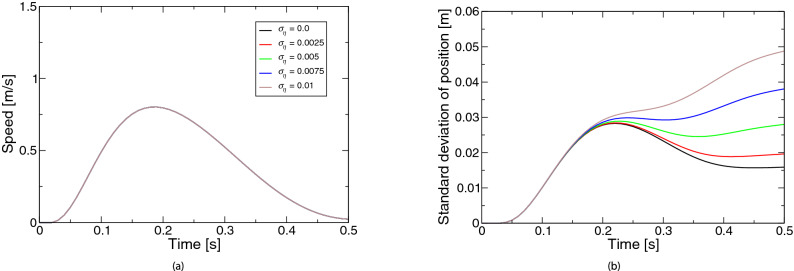
Simulated movements based on the extended LQG for the coefficient $\sigma _\eta$ of internal estimation noise: (**a**) speed, (**b**) positional variability. The legend of the subfigure (**b**) is the same as the subfigure (**a**).

Figure 4 represents the simulated movements based on the extended LQG for the coefficient $\sigma _\xi$ of the state noise. Figure 4a shows the speed profiles. The profiles were mostly bell-shaped and largely remained unchanged by the state noise. This characteristic was also confirmed for state-independent observation noise (Fig. 5a) and internal estimation noise (Fig. 6a). The scaling factor of the state-dependent observation noise in these results was zero ($\sigma _d = 0$). Although the resulting graph is not shown, the velocity peak appears slightly later when the scaling factor is not zero. Figure 4b shows the positional variability. As the state noise became larger, the variability increased over the movement. On the other hand, the variability in the state-independent observation noise and internal estimation noise did not change almost from the beginning to the middle of the movement (Figs. 5b, 6b).

## Discussion

I obtained update equations to efficiently compute the expected value and variance based on the extended LQG. Using the update equations, I computed the profiles of simulated movement speed and positional variability for various amplitudes of noises in a point-to-point reaching movement. The speed peak was changed by the control-dependent noise and state-dependent observation noise. The positional variability changes for various noises, and the period during which the variability changed differed with the noise type. The results show that multiplicative noise is an important factor in characterizing the profiles of positional variability and movement speed and that additive noise is an important factor in characterizing the profiles of positional variability. These characteristics would be helpful in how motor control based on extended LQG appears in the presence of various disturbances.

Stochastic optimal control has been studied to explain the characteristics of human upper-arm reaching movement. In particular, the extended LQG framework has been verified for movement tasks, including various disturbances^5,8,9,11^. The disturbances can be treated as noise in the extended LQG. However, in the previous studies, the expected value and variance of the optimal movement were computed based on the Monte Carlo method. The Monte Carlo method computes the expected value by repeating many trials and requires much computation time. In this study, the updated equations provided an efficient computation of expected value and variance. Efficient computation in stochastic optimal control based on the extended LQG would contribute to the elucidation of motor control under various disturbances.

## Methods

I computed the movement speed and positional variability based on the extended LQG^8^ for various amplitudes of noises. Instead of using Monte Carlo method, I obtained update equations to compute the mean and variance of state variables.

### Framework of extended LQG

The linear movement dynamic system in discrete time *t* is given as:

1 $$\begin{aligned} \varvec{x}_{t+1} \, = & \quad {} A\varvec{x}_t + B\varvec{u}_t + \varvec{\xi }_t + \sum _{i=1}^c\varepsilon _t^i C_i\varvec{u}_t \end{aligned}$$

where *A* and *B* are the dynamic system matrices, $\varvec{x}$ is the state variables, and $\varvec{u}$ is the control signal. State noise $\varvec{\xi }$ is state-independent noise which is Gaussian with mean 0 and covariance $\mathbf{\Omega ^\xi } \ge 0$. The noise $\varepsilon$ related to control-dependent noise is Gaussian with mean 0 and covariance $\mathbf{\Omega }^\varepsilon = {\textbf{I}}$. *C* is the scaling matrix for control-dependent noise. The matrix *C* is *BF*, where *F* is the scaling factor. It is already known that the initial state is mean $\widehat{\varvec{x}}_{1}$ and covariance $\mathbf{\Sigma }_{1}$.

The feedback system is as given below:

2 $$\begin{aligned} \varvec{y}_{t} \, = & \quad {} H\varvec{x}_t + \varvec{\omega }_t + \sum _{i=1}^d\epsilon _t^iD_i\varvec{x}_t \end{aligned}$$

where $\varvec{y}$ is the feedback signal and *H* is the observation matrix. State-independent observation noise $\varvec{\omega }$ is Gaussian with mean 0 and covariance $\mathbf{\Omega ^\omega } \ge 0$. The noise $\epsilon$ related to state-dependent observation noise is Gaussian with mean 0 and covariance $\mathbf{\Omega }^\epsilon = {\textbf{I}}$. *D* is the scaling matrix for state-dependent observation noise.

The state estimate $\widehat{\varvec{x}}$ is calculated as given below:

3 $$\begin{aligned} \widehat{\varvec{x}}_{t+1} \, = & \quad {} A\widehat{\varvec{x}}_t + B\varvec{u}_t + K_t(\varvec{y}_t - H\widehat{\varvec{x}}_t)+\varvec{\eta }_t \end{aligned}$$

where *K* is the filter gain matrix and internal estimation noise $\varvec{\eta }$ is Gaussian noise with mean 0 and covariance $\mathbf{\Omega ^\eta } \ge 0$.

Cost per step is calculated as given below:

4 $$\begin{aligned} \varvec{x}_t^{\mathsf {T}} Q_t\varvec{x}_t + \varvec{u}_t^{\mathsf {T}} R\varvec{u}_t \end{aligned}$$

where *Q* is the matrix of the state cost and *R* is the matrix of the control cost.

The optimal control $\varvec{u}$ is recursively computed in the opposite direction over a period of time, as follows:

5 $$\begin{aligned} \varvec{u}_t \, = & \quad {} -L_t\widehat{\varvec{x}}_t \end{aligned}$$

6 $$\begin{aligned} L_t \, = & \quad {} \left\{ R+B^{\mathsf {T}} S_{t+1}^{{\textbf {x}}}B + \sum _i C_i^{\mathsf {T}}(S_{t+1}^{{\textbf {x}}}+S_{t+1}^{{\textbf {e}}})C_i\right\} ^{-1}B^{\mathsf {T}} S_{t+1}^{{\textbf {x}}}A\nonumber \\ S_t^{{\textbf {x}}} \, = & \quad {} Q_t + A^{\mathsf {T}} S_{t+1}^{{\textbf {x}}}(A-BL_t)+\sum _i D_i^{\mathsf {T}} K_t^{\mathsf {T}} S_{t+1}^{{\textbf {e}}}K_tD_i\nonumber \\ S_t^{{\textbf {e}}} \, = & \quad {} A^{\mathsf {T}} S_{t+1}^{{\textbf {x}}}BL_t + (A-K_tH)^{\mathsf {T}} S_{t+1}^{{\textbf {e}}}(A-K_tH)\nonumber \\ s_t \, = & \quad {} \mathrm {tr}\left\{ S_{t+1}^{{\textbf {x}}}\mathbf{\Omega ^{\xi }} + S_{t+1}^{{\textbf {e}}}\left( \mathbf{\Omega ^\xi } + \mathbf{\Omega ^\eta } + K_t\mathbf{\Omega ^\omega } K_t^{\mathsf {T}}\right) \right\} + s_{t+1} \end{aligned}$$

where *L* is the control gain matrix. It is initialized as follows: $S_n^{{\textbf {x}}} = Q_n$, $S_n^{{\textbf {e}}} = 0$, and $s_n = 0$. Total expected cost is calculated as follows:

7 $$\begin{aligned} \widehat{\varvec{x}}_{1}^{\mathsf {T}}S_1^{\textbf {x}}\widehat{\varvec{x}}_{1} + \mathrm {tr}\left\{ \left( S_1^{{\textbf {x}}} + S_1^{{\textbf {e}}}\right) \mathbf{\Sigma }_{1}\right\} + s_1. \end{aligned}$$

The optimal filter is recursively computed forward in time as follows:

8 $$\begin{aligned} \widehat{\varvec{x}}_{t+1} \, = & \quad {} (A-BL_t)\widehat{\varvec{x}}_t + K_t(\varvec{y}_t - H\widehat{\varvec{x}}_t) + \varvec{\eta }_t \end{aligned}$$

9 $$\begin{aligned} K_t \, = & \quad {} A\mathbf{\Sigma }_t^{{\textbf {e}}}H^{\mathsf {T}} \left\{ H\mathbf{\Sigma }_t^{\textbf {e}}H^{\mathsf {T}} + \mathbf{\Omega ^\omega } + \sum _i D_i(\mathbf{\Sigma }_t^{\textbf {e}} +\mathbf{\Sigma }_t^\mathbf {{\widehat{x}}}+ \mathbf{\Sigma }_t^\mathbf {{\widehat{x}}e} + \mathbf{\Sigma }_t^\textbf{e} {\mathbf{\widehat{x}}})D_i^{\mathsf {T}} \right\} ^{-1}\nonumber \\ \mathbf{\Sigma }_{t+1}^{{\textbf {e}}} \, = & \quad {} \mathbf{\Omega ^\xi } + \mathbf{\Omega ^\eta } + (A-K_tH)\mathbf{\Sigma }_t^{\textbf {e}}A^{\mathsf {T}} + \sum _i C_iL_t \mathbf{\Sigma }_t^\mathbf {{\widehat{x}}}L_t^{\mathsf {T}}C_i^{\mathsf {T}}\nonumber \\ \mathbf{\Sigma }_{t+1}^{\mathbf {{\widehat{x}}}} \, = & \quad {} \mathbf{\Omega ^\eta } + K_tH\mathbf{\Sigma }_t^{\textbf {e}}A^{\mathsf {T}} + (A-BL_t)\mathbf{\Sigma }_t^\mathbf {{\widehat{x}}}(A-BL_t)^{\mathsf {T}}\nonumber \\&+ (A-BL_t)\mathbf{\Sigma }_t^\mathbf {{\widehat{x}}e}H^{\mathsf {T}}K_t^{\mathsf {T}} + K_tH\mathbf{\Sigma }_t^\textbf{e} {\mathbf{\widehat{x}}}(A-BL_t)^{\mathsf {T}} \nonumber \\ \mathbf{\Sigma }_{t+1}^{\mathbf {{\widehat{x}}e}} \, = & \quad {} (A-BL_t)\mathbf{\Sigma }_t^\mathbf {{\widehat{x}}e}(A-K_tH)^{\mathsf {T}} - \mathbf{\Omega ^\eta }. \end{aligned}$$

It is initialized as follows: $\mathbf{\Sigma }_{1}^{{\textbf {e}}} = \mathbf{\Sigma }_{1},~\mathbf{\Sigma }_{1}^{\mathbf {{\widehat{x}}}} = {\widehat{\varvec{x}}}_1{\widehat{\varvec{x}}}_1^{\mathsf {T}},~\mbox{and}~\mathbf{\Sigma }_{1}^{\mathbf {{\widehat{x}}e}} = 0$.

### Mean and variance of state variables

The update equations used to compute the mean and variance of state variables were obtained from Eq. (1) and the following equations.

10 $$\begin{aligned} \widehat{\varvec{x}}_{t+1} \, = & \quad {} (A-BL_t)\widehat{\varvec{x}}_t + K_tH\varvec{e}_t + K_t\varvec{\omega }_t + \varvec{\eta }_t + \sum _i\epsilon ^i_tK_tD_i(\varvec{e}_t + \widehat{\varvec{x}}_t) \end{aligned}$$

11 $$\begin{aligned} \varvec{e}_{t+1} \, = & \quad {} (A-K_tH)\varvec{e}_t+\varvec{\xi }_t - K_t\varvec{\omega }_t - \varvec{\eta }_t - \sum _i\varepsilon ^i_tC_iL_t\widehat{\varvec{x}}_t - \sum _i \epsilon ^i_tK_tD_i(\varvec{e}_t + \widehat{\varvec{x}}_t) \end{aligned}$$

where the estimation error $\varvec{e}_t$ is $\varvec{x}_{t} - \widehat{\varvec{x}}_{t}$. Equations (10) and (11) are derived from Eqs. (2) and (3), and Eqs. (1) and (10), respectively.

The mean of state variables can be computed sequentially by applying the following update equations.

12 $$\begin{aligned} \text{ E }[\varvec{x}_{t+1}] \, = & \quad {} A\text{ E }[\varvec{x}_t] - BL_t\text{ E }[\widehat{\varvec{x}}_t] \end{aligned}$$

13 $$\begin{aligned} \text{ E }[\widehat{\varvec{x}}_{t+1}] \, = & \quad {} (A-BL_t)\text{ E }[\widehat{\varvec{x}}_t] + K_tH\text{ E }[\varvec{e}_t] \end{aligned}$$

14 $$\begin{aligned} \text{ E }[\varvec{e}_{t+1}] \, = & \quad {} (A-K_tH)\text{ E }[\varvec{e}_t] \end{aligned}$$

The variance of state variables can be computed sequentially by using the following update equations:

15 $$\begin{aligned} \text{ var }[\varvec{x}_{t+1}] \, = & \quad {} A\text{ var }[\varvec{x}_t]A^{\mathsf {T}} \nonumber \\- & {} A\text{ cov }[\varvec{x}_t,~\widehat{\varvec{x}}_t]L_t^{\mathsf {T}}B^{\mathsf {T}}\nonumber \\- & {} BL_t\text{ cov }[\widehat{\varvec{x}}_t,~\varvec{x}_t]A^{\mathsf {T}}\nonumber \\+ & {} BL_t\text{ var }[\widehat{\varvec{x}}_t]L_t^{\mathsf {T}}B^{\mathsf {T}}\nonumber \\+ & {} \mathbf{\Omega ^\xi } + \sum _{i} C_iL_t \mathbf{\Sigma }_t^\mathbf {{\widehat{x}}} L_t^{\mathsf {T}}C_i^{\mathsf {T}} \end{aligned}$$

16 $$\begin{aligned} \text{ cov }[\varvec{x}_{t+1},~\widehat{\varvec{x}}_{t+1}] \, = & \quad {} A\text{ cov }[\varvec{x}_t,~\widehat{\varvec{x}}_t](A-BL_t)^{\mathsf {T}} \nonumber \\+ & {} A\text{ cov }[\varvec{x}_t,~\varvec{e}_t]H^{\mathsf {T}}K_t^{\mathsf {T}}\nonumber \\- & {} BL_t\text{ var }[\widehat{\varvec{x}}_t](A-BL_t)^{\mathsf {T}}\nonumber \\- & {} BL_t \text{ cov }[\widehat{\varvec{x}}_t,~\varvec{e}_t]H^{\mathsf {T}}K_t^{\mathsf {T}} \end{aligned}$$

17 $$\begin{aligned} \text{ var }[\widehat{\varvec{x}}_{t+1}] \, = & \quad {} (A-BL_t) \text{ var }[\widehat{\varvec{x}}_t](A-BL_t)^{\mathsf {T}} \nonumber \\+ & {} (A-BL_t)\text{ cov }[\widehat{\varvec{x}}_t,~\varvec{e}_t]H^{{\mathsf {T}}}K_t^{{\mathsf {T}}}\nonumber \\+ & {} K_tH\text{ cov }[\varvec{e}_t,~\widehat{\varvec{x}}_t](A-BL_t)^{{\mathsf {T}}}\nonumber \\+ & {} K_tH\text{ var }[\varvec{e}_t]H^{\mathsf {T}}K_t^{\mathsf {T}}\nonumber \\+ & {} K_t\mathbf{\Omega ^{\omega }}K_t^{\mathsf {T}} + \mathbf{\Omega ^\eta }\nonumber \\+ & {} \sum _i K_tD_i\mathbf{\Sigma }_t^{\textbf {x}}D_i^{\mathsf {T}}K_t^{\mathsf {T}} \end{aligned}$$

18 $$\begin{aligned} \text{ cov }[\varvec{x}_{t+1},~\varvec{e}_{t+1}] \, = & \quad {} A\text{ cov }[\varvec{x}_t,~\varvec{e}_t](A-K_tH)^{\mathsf {T}} \nonumber \\- & {} BL_t\text{ cov }[\widehat{\varvec{x}}_t,~\varvec{e}_t](A-K_tH)^{\mathsf {T}}\nonumber \\+ & {} \mathbf{\Omega }^{\xi } + \sum _iC_iL_t\mathbf{\Sigma }_t^\mathbf {{\widehat{x}}}L_t^{\mathsf {T}}C_i^{\mathsf {T}} \end{aligned}$$

19 $$\begin{aligned} \text{ cov }[\widehat{\varvec{x}}_{t+1},~\varvec{e}_{t+1}] \, = & \quad {} (A-BL_t)\text{ cov }[\widehat{\varvec{x}}_t,~\varvec{e}_t](A-K_tH)^{\mathsf {T}} \nonumber \\+ & {} K_tH\text{ var }[\varvec{e}_t](A-K_tH)^{{\mathsf {T}}}\nonumber \\- & {} K_t\mathbf{\Omega }^{\omega }K_t^{{\mathsf {T}}} - \mathbf{\Omega }^{\eta } - \sum _iK_tD_i\mathbf{\Sigma }_t^{\textbf {x}}D_i^{\mathsf {T}}K_t^{\mathsf {T}} \end{aligned}$$

20 $$\begin{aligned} \text{ var }[\varvec{e}_{t+1}] \, = & \quad {} (A-K_tH)\text{ var }[\varvec{e}_t](A-K_tH)^{\mathsf {T}} \nonumber \\+ & {} \mathbf{\Omega ^\xi } + K_t\mathbf{\Omega ^\omega } K_t^{\mathsf {T}} + \mathbf{\Omega ^\eta }\nonumber \\+ & {} \sum _iC_iL_t\mathbf{\Sigma }_t^\mathbf {{\widehat{x}}}L_t^{\mathsf {T}}C_i^{\mathsf {T}} + \sum _iK_tD_i\mathbf{\Sigma }_t^{\textbf {x}}D_i^{\mathsf {T}}K_t^{\mathsf {T}} \end{aligned}$$

### Application to the reaching movement

The simulated movement task was a single joint reaching movement similar to the study^8^. The movement was replaced with a translational point-to-point reaching movement for simplicity. The movement duration $t_{end}$ was set to 0.5 s. The starting position was 0 m, while the target position $p^*$ was 0.2 m.

**Table 1:** Parameters of the movement dynamic system.

Parameter	Value
$\Delta t$	0.01 (s)
*m*	1.0 (kg)
$\tau _1$	40.0 (ms)
$\tau _2$	40.0 (ms)
*c*	1

The matrices of the dynamic system in the translational point-to-point reaching movement are depicted below:

$$\begin{aligned} A \, = & \quad {} \begin{bmatrix} 1 &{} \Delta t &{} 0 &{} 0 &{} 0\\ 0 &{} 1 &{} \Delta t/m &{} 0 &{} 0\\ 0 &{} 0 &{} 1-\Delta t/\tau _2 &{} \Delta t/\tau _2 &{} 0\\ 0 &{} 0 &{} 0 &{} 1-\Delta t/\tau _1 &{} 0\\ 0 &{} 0 &{} 0 &{} 0 &{} 1\\ \end{bmatrix}\\ B \, = & \quad {} \begin{bmatrix} 0\\ 0\\ 0\\ \Delta t/\tau _1\\ 0\\ \end{bmatrix} \\ C_1 \, = & \quad {} B\sigma _c \end{aligned}$$

where $\Delta t$ is the time step, *m* is the point mass, and $\tau _1$ and $\tau _2$ are the time constants (Table 1). The state $\varvec{x}$ of the system is $[p(t),~{\dot{p}}(t),~f(t),~{\dot{f}}(t),~p^*]^{\mathsf {T}}$, where *p* is the position and *f* is the force. The initial state was $\widehat{\varvec{x}}_{1} = [0,~0,~0,~0,~p ^*]^{\mathsf {T}}$ and $\mathbf{\Sigma }_{1} = 0$.

**Table 2:** Parameters of the feedback system.

Parameter	Value
$\omega _p$	0.02
$\omega _v$	0.2
$\omega _f$	1.0
*d*	3

The matrices of the feedback system are depicted below:

$$\begin{aligned} H \, = & \quad {} \begin{bmatrix} 1 &{} 0 &{} 0 &{} 0 &{} 0\\ 0 &{} 1 &{} 0 &{} 0 &{} 0\\ 0 &{} 0 &{} 1 &{} 0 &{} 0\\ \end{bmatrix} \\ D_1 \, = & \quad {} \sigma _d \begin{bmatrix} \omega _p &{} 0 &{} 0 &{} 0 &{} 0\\ 0 &{} 0 &{} 0 &{} 0 &{} 0\\ 0 &{} 0 &{} 0 &{} 0 &{} 0\\ \end{bmatrix} \\ D_2 \, = & \quad {} \sigma _d \begin{bmatrix} 0 &{} 0 &{} 0 &{} 0 &{} 0\\ 0 &{} \omega _v &{} 0 &{} 0 &{} 0\\ 0 &{} 0 &{} 0 &{} 0 &{} 0\\ \end{bmatrix} \\ D_3 \, = & \quad {} \sigma _d \begin{bmatrix} 0 &{} 0 &{} 0 &{} 0 &{} 0\\ 0 &{} 0 &{} 0 &{} 0 &{} 0\\ 0 &{} 0 &{} \omega _f &{} 0 &{} 0\\ \end{bmatrix} \end{aligned}$$

where $\sigma _d$ is the scaling factor. Table 2 shows the parameters of the feedback system.

The state noise, state-independent observation noise, and internal estimation noise have covariances $\mathbf{\Omega ^{\xi }} = \sigma _\xi ^2{\textbf{I}}$, $\mathbf{\Omega ^{\omega }} = \left( \sigma _\omega \mathrm {diag}[\omega _p,~\omega _v,~\omega _f]\right) ^2$, and $\mathbf{\Omega ^{\eta }} = \sigma _\eta ^2{\textbf{I}}$, respectively. The cost matrices are $R = r$, $Q_{1,~\ldots ,~n-1} = 0$, and $Q_n = {\textbf {p}}{\textbf {p}}^{\mathsf {T}} + {\textbf {v}}{\textbf {v}}^{\mathsf {T}} + {\textbf {f}}{\textbf {f}}^{\mathsf {T}}$, where ${\textbf {p}} = [1,~0,~0,~0,~-1]^{\mathsf {T}}$, ${\textbf {v}} = [0,~0.2,~0,~0,~0]^{\mathsf {T}}$, and ${\textbf{f}} = [0, 0, 0.02, 0, 0]^{\mathsf{T}}$.

**Table 3:** The basic parameter set.

Parameter	Value
$\sigma _c$	1.5
$\sigma _\xi$	0.0
$\sigma _\omega$	0.0
$\sigma _d$	0.0
$\sigma _\eta$	0.0
*r*	1e−10

The simulated movements were obtained by changing each value of $\sigma _c$, $\sigma _\xi$, $\sigma _\omega$, $\sigma _d$, $\sigma _\eta$, and *r* in the basic parameter set (Table 3). The optimization was completed when the absolute value of the relative change in the total expected cost became less than the convergence tolerance ($1.0\times 10^{-15}$).
